# Assisted Living Administrators’ Mental and Emotional Health During the COVID-19 Pandemic

**DOI:** 10.1093/geroni/igab046.3662

**Published:** 2021-12-17

**Authors:** Jaclyn Winfree, Ozcan Tunalilar, Jason Kyler-Yano, Serena Hasworth, Paula Carder

**Affiliations:** 1 Portland State University, Portland, Oregon, United States; 2 Portland State University, Portland State University, Oregon, United States; 3 OHSU-PSU School of Public Health, Portland, Oregon, United States

## Abstract

Little is known about assisted living (AL) administrators’ mental and emotional health, particularly during a global pandemic in which most of their residents are highly vulnerable to infection, hospitalization, and death. Considering that administrator turnover and burnout have been associated with negative outcomes such as decreased quality of resident care, low staff morale, and reduced financial solvency, this study examined how AL administrators described their mental and emotional state throughout the first year of the COVID-19 pandemic. Using thematic analysis, our team coded 18 qualitative interviews conducted from May-August 2021. The themes included declining physical health due to stress, feelings of inadequacy and self-doubt, and increased burnout. Many administrators described increased staffing challenges as directly impacting their daily stress levels. Some administrators described feeling guilty and doubting their interpretation or implementation of regulations, particularly in incidents that further distanced residents from peers and loved ones. A few administrators described their disposition or personality changing due to what they experienced during the pandemic. One administrator stated, “I'm not an anxiety person, but I feel anxiety about a lot of things. In fact, my doctor has talked to me about starting some medications to help with that.” Multiple administrators made comments such as, “I don't know that there could be a more stressful position than executive director of assisted living…the COVID pandemic reinforced that. This is rough.” Understanding AL administrators’ mental and emotional health during a public health crisis allows for understanding, supporting, and retaining critical leaders in long-term care communities.

